# Synthesis of New Series of 2-*C*-(β-D-glucopyranosyl)-Pyrimidines and Their Evaluation as Inhibitors of Some Glycoenzymes

**DOI:** 10.3390/molecules25030701

**Published:** 2020-02-06

**Authors:** Eszter Szennyes, Gyöngyi Gyémánt, László Somsák, Éva Bokor

**Affiliations:** 1 Department of Organic Chemistry, University of Debrecen, P.O. Box 400, H-4002 Debrecen, Hungary; szeszterke11@gmail.com; 2 Department of Inorganic and Analytical Chemistry, University of Debrecen, P.O. Box 400, H-4002 Debrecen, Hungary; gyemant@science.unideb.hu

**Keywords:** *C*-Glucopyranosyl derivative, pyrimidine, amidine, glycoenzyme, inhibitor

## Abstract

Despite the substantial interest in *C*-glycosyl heterocycles as mimetics of biologically active native glycans, the appearance of *C*-glycopyranosyl derivatives of six-membered heterocycles, both in synthetic and biological contexts, is rather scarce. As part of our ongoing research program aimed at preparing hitherto barely known 2-*C*-glycopyranosyl pyrimidines, the goal of the present study was to synthesize new 5-mono- and multiply substituted derivatives of this compound class. Thus, 2-*C*-(β-D-glucopyranosyl)-5,6-disubstituted-pyrimidin-4(3*H*)-ones and 4-amino-2-*C*-(β-D-glucopyranosyl)-5,6-disubstituted-pyrimidines were prepared by base-mediated cyclocondensations of *O*-perbenzylated and *O*-unprotected *C*-(β-D-glucopyranosyl) formamidine hydrochlorides with methylenemalonic acid derivatives. The 2-*C*-(β-D-glucopyranosyl)-5-substituted-pyrimidines were obtained from the same amidine precursors upon treatment with vinamidinium salts. The deprotected derivatives of these pyrimidines were tested as inhibitors of some glycoenzymes. None of them showed inhibitory activity towards glycogen phosphorylase and α- and β-glucosidase enzymes, but some members of the sets exhibited moderate inhibition against bovine liver β-galactosidase.

## 1. Introduction

*C*-Glycopyranosyl heterocycles [1] are among the widely investigated groups of sugar-based small molecules. The intense interest in such compounds is primarily due to their possible use as glycomimetics [1,2,3]. The hydrolitically stable C-C linkage between the glycon and the heterocyclic aglycon part, and the ability of the heteroaromatic moiety to strengthen the binding by diverse interactions (e.g., hydrogen bonds, van der Waals interactions, π-π stackings, and coordination to metal ions) to target biomolecules, along with some general advantages derived from the presence of the sugar component (e.g., enhancement of the solubility, the possibility of targeting carbohydrate binding proteins), make these compounds very attractive in drug design [4].

Within this compound class, the most commonly represented ones are *C*-glycopyranosyl derivatives of five-membered heterocycles, possessing a large variety of biological effects [1]. On the other hand, six-membered *C*-glycopyranosyl heterocycles have received much less attention [1]. This appears to be surprising given that their *C*-glycofuranosyl variants, as analogues of nucleosides, belong to an intensively studied class of sugar conjugates [5]. This general tendency also applies to *C*-glycosyl pyrimidines: while a great number of *C*-glycofuranosyl pyrimidines are known [6,7,8,9,10,11], *C*-glycopyranosyl analogues are scarcely found in the literature.

For the formation of 2-*C*-glycopyranosyl pyrimidines, only one example was described [12], wherein a Minisci type radical glycosylation of a protonated pyrimidine resulted in a 2-(3′,4′-di-*O*-benzoyl-2′-deoxy-β-D-ribopyranosyl)-pyrimidine together with the corresponding 4-*C*-glycopyranosylated isomer in 3:7 ratio. Some 5-*C*-glycopyranosyl pyrimidines were produced by ring-closures of *C*-glycopyranosylated enaminoketones with guanidine or acetamidine [13,14]. In addition, series of 4-*C*-, 6-*C*-, and 4,6-bis-*C*-glycosyl dihydropyrimidines were obtained from *C*-glycopyranosyl formaldehydes or β-ketoesters by three-component Biginelli-type cyclisations [15,16].

**Figure 1:**
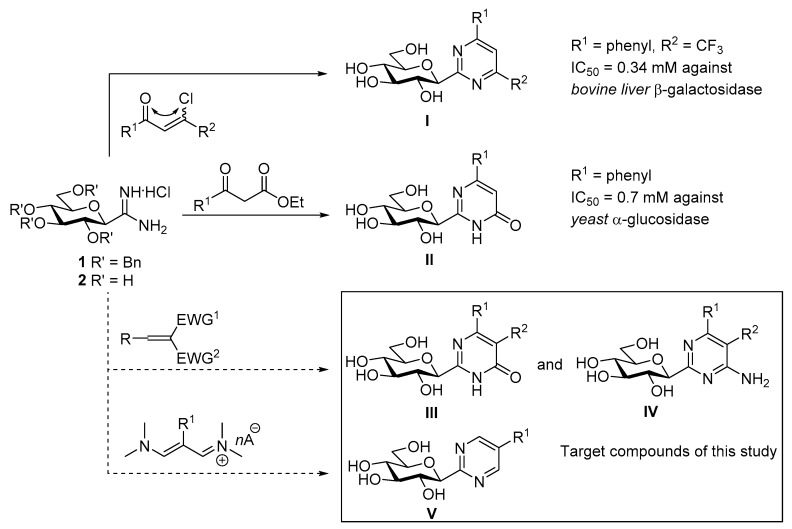
Recent syntheses of 2-*C*-glucopyranosyl pyrimidines yielding biologically active derivatives and the target compounds of this study.

Recently, as part of a systematic study on the syntheses of 2-*C*-glycopyranosyl pyrimidines (e.g., **I** and **II** in Figure 1), we published their first general synthesis from the corresponding *O*-perbenzylated (**1**) or *O*-unprotected *C*-glucopyranosyl formamidines (**2**) as well as in a one-pot three-step transformation of *O*-peracylated glycopyranosyl cyanides [17]. Some members of **I** and **II** exhibited moderate inhibition of some glycosidase enzymes [17], however, each proved inactive against glycogen phosphorylase [17]. Although these biological effects are not outstanding, these are the first investigations to reveal potential utilities of this novel compound class.

As a continuation of these studies, in this paper, we disclose the preparation of 4,5,6-tri- and 5-monosubstituted 2-*C*-glucopyranosyl pyrimidines (**III**, **IV** and **V**, respectively) by the reaction of amidines **1** and **2** with methylenemalonic acid derivatives and vinamidinium salts, respectively, and the evaluation of the resulting heterocycles as inhibitors of glycoenzymes.

## 2. Results and Discussion

### 2.1. Syntheses

For the synthesis of the target trisubstituted 2-*C*-glucopyranosyl pyrimidines, the ring-closures of amidine hydrochloride **1** [18,19] with methylenemalonic acid derivatives **3–7** were investigated first (Table 1). Treatment of **1** with compounds **3**–**7** in the presence of NaOMe in MeOH at 0 °C gave the desired pyrimidines **10a–f**, respectively, in good yields. In the reaction of **1** with ethyl 2-cyano-3-ethoxyacrylate **4**, the nucleophilic amidine attacked both the cyano and the ester groups of the reagent. Thus, this cyclocondensation led to the formation of a mixture of ethyl 4-amino-pyrimidine-5-carboxylate **10b** and 6-oxo-1,6-dihydropyrimidine-5-carbonitrile **10c**. Surprisingly, the same reaction of **1** with ethyl 2-cyano-2-phenylacrylate **7** afforded only one product **11f**, derived from a ring-closure involving the ester group of the reagent. 

For the *O*-debenzylation of the new 2-glucosyl pyrimidines **10a–f**, catalytic hydrogenolysis in an acidified EtOAc-EtOH solvent mixture at ambient temperature was attempted. Under the applied reductive conditions, the deprotection of compounds **10b** and **10d** was smoothly affected to get the test compounds **11b** and **11d**, respectively, in acceptable yields. Unfortunately, pyrimidines **10a**,**c**,**e**,**f** with a 5-CN substituent remained intact under the same conditions. This might be due to a poisoning of the catalyst, caused by the coordination of the cyano group to the palladium.

In order to avoid the critical deprotection in the last step of the synthesis, the preparation of the unprotected pyrimidines **11** was also examined in a reversed sequence, wherein the formamidine salt **2**, obtained from **1** by hydrogenolytic *O*-debenzylation [17], was cyclized with the corresponding methylenemalonic acid derivatives **3–7** (Table 1). The ring-closure of **2** with compounds **3–7** proceeded similarly to that of amidine salt **1**, providing each target test compound **11a–f** in moderate to good yields.

**Scheme 1:**
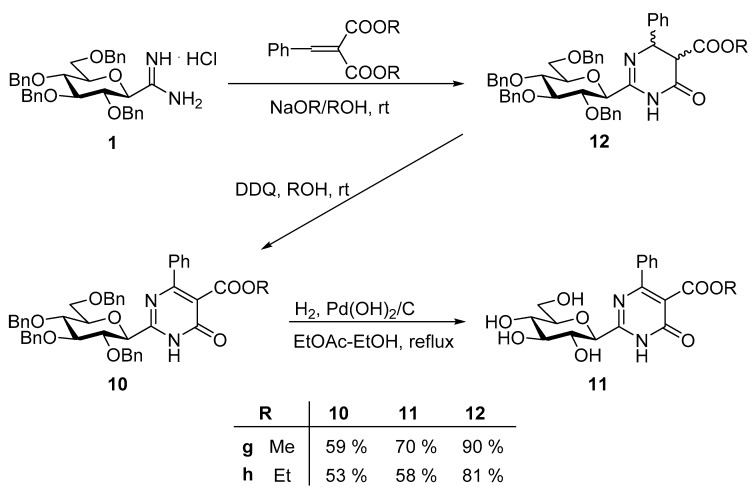
Synthesis of alkyl 2-(β-D-glucopyranosyl)-4-phenyl-6-oxo-1,6-dihydropyrimidine-5-carboxylates.

The cyclocondensations of amidine salts **1** and **2** with dialkyl benzylidenemalonates **8** and **9**, under the same ring-closing conditions used for compounds **3–7**, did not directly provide the expected pyrimidinone derivatives **10g**,**h** and **11g**,**h** (Table 1). Similarly to a literature example [20], compounds **8** and **9**, when cyclized with **2**, furnished 6-oxo-1,4,5,6-tetrahydropyrimidines **12** (Scheme 1). Our attempts to achieve the spontaneous oxidation of compounds **12g**,**h** to get **10g**,**h** by using prolonged reaction times or higher temperatures, were unsuccessful. Finally, the transformation of **12g**,**h** into **10g**,**h** was carried out by applying DDQ as an oxidant in an additional step. The removal of the *O*-benzyl protecting groups of **12g**,**h** was then performed by hydrogenolysis over Pd(OH)_2_ to get the final products **11g**,**h** in good yields.

The formation of 2-*C*-glucopyranosyl-5-substituted-pyrimidines was also envisaged starting from the same carbohydrate precursors **1** and **2**. To this end, NaOMe-mediated cyclisations of compounds **1** and **2** with vinamidinium salts **13–16** were accomplished to get the desired 2,5-disubstituted heterocycles **17** and **18**, respectively, in good to high yields (Table 2). Compound **18a** was prepared both by the ring-closure of **1** with **13**, followed by a BCl_3_-mediated *O*-debenzylation of the resulting pyrimidine **17a**, and by a reversed debenzylation-cyclisation sequence **1**→**2**→**18a**. In terms of the overall yields of **18a**, the latter route proved to be more efficient (51% for **1**→**17a**→**18a** vs. 80% for **1**→**2**→**18a**). By applying this second synthetic pathway, high-yielding preparation of the test compounds **18b** and **18c** was also smoothly achieved (Table 2).

**Scheme 2:**
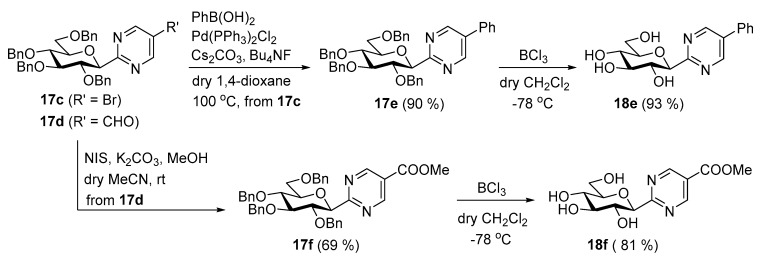
Synthesis of further 2-(β-D-glucopyranosyl)-5-substituted-pyrimidines.

In addition, further transformations of compounds **17c** and **17d** were carried out to get additional 2-*C*-glucopyranosyl-5-substituted-pyrimidines (Scheme 2). A Pd(PPh_3_)_2_Cl_2_-catalyzed cross-coupling of 5-bromopyrimidine **17c** with phenylboronic acid furnished 5-phenylpyrimidine **17e** in excellent yield, while the oxidation of 5-formylpyrimidine **17d** with NIS in the presence of K_2_CO_3_ and MeOH resulted in methyl pyrimidine-5-carboxylate **17f** in good yield. Finally, the cleavage of the *O*-benzyl protecting groups of **17e**,**f** was performed with BCl_3_ to obtain the test compounds **18e**,**f** in high yields.

### 2.2. Enzyme Inhibition Studies

The new unprotected compounds **11** and **18** were tested as inhibitors of some glycoenzymes. Similarly to the previously tested 2-*C*-glucopyranosyl pyrimidines (**I** and **II** in Figure 1) [17], none of them exhibited inhibition against rabbit muscle glycogen phosphorylase *b* (rmGPb) and almond β-glucosidase. 

While 2-(β-D-glucopyranosyl)-6-phenylpyrimidin-4(3*H*)-one **19** was earlier shown to be a submillimolar inhibitor of yeast α-glucosidase (IC_50_ = 0.7 mM) [17], the new analogs **11** had negligible effects against this enzyme.

Depending on the substitution pattern of the pyrimidine ring, varied inhibitory potencies of compounds **11** and **19** were observed towards bovine liver β-galactosidase (Table 3). The enzyme kinetic data of the comparable pairs **11a** and **11e**, **11c** and **11f**, and **11d** and **11h** clearly indicated the beneficial effect of the presence of a phenyl substituent at the C-6 position of the pyrimidine ring: while compounds **11a**, **11c**, and **11d** did not inhibit the β-galactosidase, their phenyl-substituted counterparts **11e**, **11f**, and **11h**, respectively, displayed a weak but noticeable inhibition in similar mM concentration ranges. The inhibitory activity of **11f-h** in comparison to that of **19** showed that the introduction of a cyano group into the C-5 position of the heterocycle did not cause any significant effect on the potency (**19** vs. **11f**), but switching to the ester groups resulted in some strengthening of the inhibition (**19** vs. **11g** and **11h**). A similar slight improvement was also observed in the pair **11a** and **11b**. Compound **11h**, bearing both the phenyl and the ester substituent, proved to be the best inhibitor of the series, displaying submillimolar inhibitory effect against this β-galactosidase enzyme.

Among the 2-(β-D-glucopyranosyl)-5-substituted-pyrimidines, the unsubstituted **18a** and the 5-halogen-substituted heterocycles **18b**,**c** proved to be inactive, while pyrimidines, having the phenyl (**18e**) and the methyl ester (**18f**) group, showed weak inhibition against the β-galactosidase enzyme (Table 3). Although compounds **18e** and **18f** had no significant effects against this enzyme, their moderate activities indicated that the introduction of these substituents, not only at position 6 but also at 5 of the pyrimidine ring could also be advantageous. 

## 3. Experimental 

### 3.1. Syntheses

#### 3.1.1. General Methods

Optical rotations were measured on a Jasco P-2000 polarimeter (Jasco, Easton, MD, USA) at rt, and the data were calculated as an average of three parallel measurements. NMR spectra were recorded with Bruker DRX360 (360/90 MHz for ^1^H/^13^C) and Bruker DRX400 (400/100 MHz for ^1^H/^13^C) spectrometers. Chemical shifts are referenced to Me_4_Si (^1^H) or to the residual solvent signals (^13^C). MS spectra were obtained by a Bruker Micro TOF-Q (ESI-MS) or a Bruker maXis II (ESI-HRMS) spectrometer. For TLC analysis, DC Alurolle Kieselgel 60 F_254_ plates (Merck) were used and the spots were visualized under UV light and by gentle heating. For column chromatographic purification, Kieselgel 60 silica gel (Molar Chemicals, particle size 63–200 µm) was used. Anhydrous MeOH was dried by distillation over Mg turnings and iodine. Anhydrous EtOH was purchased from Molar Chemicals and used as received. 2-(Ethoxymethylene)malononitrile (**3**), ethyl 2-cyano-3-ethoxyacrylate (**4**), and diethyl 2-(ethoxymethylene)malonate (**5**) were commercially available chemicals (Merck). *C*-(2,3,4,6-Tetra-*O*-benzyl-β-D-glucopyranosyl)formamidine hydrochloride (**1**) [18,19], *C*-(β-D-glucopyranosyl)formamidine hydrochloride (**2**) [17], 2-benzylidenemalononitrile (**6**) [21], 2-cyano-3-phenylacrylate (**7**) [22], dimethyl and diethyl benzylidenemalonate (**8** and **9** [22,23], respectively), 1,3-bis(dimethylamino)trimethinium perchlorate (**13**) [24], 2-chloro-1,3-bis(dimethylamino)trimethinium hexafluorophosphate (**14**) [25], and 2-dimethylaminomethylene-1,3-bis(dimethylimonio)propane diperchlorate (**16**) [26] were prepared according to published procedures.

#### 3.1.2. General Procedure 1 for the Synthesis of 2-(β-D-Glucopyranosyl)-pyrimidines (**10** or **11**) by Cyclisation of *C*-β-D-Glucopyranosyl Formamidines (**1** or **2**) with Substituted Methylenemalonic Acid Derivatives 

To a solution of the corresponding *C*-(β-D-glucopyranosyl)formamidine hydrochloride (**1** or **2**) in dry MeOH (2 mL/100 mg amidine), ~1M solution of NaOMe in dry MeOH (3 equiv.) was added at 0 °C. After stirring the reaction mixture at this temperature for 10 min, the appropriate methylenemalonic acid derivative (2 equiv.) was added. The completion of the reaction was monitored by TLC (CHCl_3_-MeOH = 9:1 and EtOAc-hexane = 1:1 in the case of *O*-perbenzylated derivatives and CHCl_3_-MeOH = 7:3 in the case of unprotected derivatives). After the disappearance of the starting amidine (**1** or **2**), the reaction mixture was neutralized with glacial acid, the solvent was evaporated under reduced pressure, and the residue was purified by column chromatography. 

#### 3.1.3. General Procedure 2 for the Synthesis of Alkyl 2-(2′,3′,4′,6′-tetra-*O*-benzyl-β-D-glucopyra-nosyl)-4-phenyl-6-oxo-1,4,5,6-tetrahydropyrimidine-5-carboxylates (**12**) by Cyclisation of *C*-β-D-Glucopyranosyl Formamidine (**1**) with Benzylidenemalonate Derivatives 

To a solution of amidine hydrochloride **1** (400 mg, 0.66 mmol) in dry MeOH or EtOH (2.5 mL/100 mg substrate), ~1M solution of sodium alkoxide in MeOH or EtOH (2 equiv.) was added and the mixture was stirred at rt for 10 min. To this mixture, the corresponding 2-benzylidenemalonate derivative (2 equiv.) was added and stirred at rt until the TLC (EtOAc-hexane = 1:2 and CHCl_3_-MeOH = 9:1) showed the complete conversion of **1** (~6 h). The reaction mixture was then neutralized with glacial acid, concentrated under diminished pressure, and the residue was purified by column chromatography.

#### 3.1.4. General Procedure 3 for the Oxidation of 1,4,5,6-Tetrahydropyrimidine Derivatives (**12**) by DDQ

A 1,4,5,6-tetrahydropyrimidine derivative (**12**) was dissolved in dry MeOH (2 mL/100 mg substrate), and DDQ (1 equiv.) was added. The reaction mixture was stirred at rt and monitored by TLC (EtOAc-hexane = 1:2). After the total consumption of the starting material (6 h), the solvent was removed in vacuo. The resulting oil was dissolved in EtOAc (30 mL) and extracted with 10% aq. solution of NaOH (5 × 10 mL). The organic phase was dried over MgSO_4_, filtered, and the solvent was evaporated under reduced pressure. The residue was purified by column chromatography.

#### 3.1.5. General Procedure 4 for the Synthesis of 2-(β-D-Glucopyranosyl)-pyrimidines (**17** or **18**) by Cyclisation of *C*-β-D-Glucopyranosyl Formamidine Hydrochlorides (**1** or **2**) and Vinamidinium Salts 

To a solution of *C*-(β-D-glucopyranosyl)formamidine hydrochloride (**1** or **2**) in dry MeOH (2 mL/100 mg amidine), ~1M solution of NaOMe in dry MeOH (2.1 equiv.) was added. The reaction mixture was stirred at rt for 10 min, then the corresponding vinamidinium salt (1.1 equiv.) was added and the stirring was continued at rt. After completion of the reaction judged by TLC (CHCl_3_-MeOH = 9:1 and EtOAc-hexane = 1:2 for benzylated compounds and CHCl_3_-MeOH = 7:3 for unprotected derivatives), the mixture was neutralized with glacial acetic acid, then the solvent was removed under diminished pressure. The residue was purified by column chromatography.

#### 3.1.6. Synthesis and Characterization of the New Compounds

*4-Amino-2-(2′,3′,4′,6′-tetra-*O*-benzyl-β-D-glucopyranosyl)-pyrimidine-5-carbonitrile* (**10a**). Prepared from compound **1** (400 mg, 0.66 mmol) and 2-(ethoxymethylene)malononitrile **3** (162 mg, 1.33 mmol) according to genereal procedure 1. Reaction time: 30 min. The title compound precipitated from the reaction mixture as a pale yellow amorphous solid. Yield: 325 mg (76%). R_f_ = 0.55 (EtOAc-hexane = 1:1); [α]_D_ = ‒1 (c 0.20, CH_2_Cl_2_); ^1^H NMR (400 MHz, CDCl_3_) δ (ppm): 8.87 (2H, br s, NH_2_), 8.33 (1H, s, H-6), 7.40–6.99 (20H, m, aromatics), 4.93, 4.89 (2 × 1H, 2d, *J* = 11.0 Hz in each, Ph*CH_2_*), 4.92, 4.69 (2 × 1H, 2d, *J* = 10.8 Hz in each, Ph*CH_2_*), 4.82, 4.74 (2 × 1H, 2d, *J* = 12.2 Hz in each, Ph*CH_2_*), 4.65, 4.22 (2 × 1H, 2d, *J* = 11.3 Hz in each, Ph*CH_2_*), 4.38 (1H, d, *J* = 9.5 Hz, H-1′), 3.94 (1H, pt, *J* = 9.5, 9.2 Hz, H-3′ or H-4′), 3.85 (1H, pt, *J* = 9.4, 9.3 Hz, H-2′ or H-3′ or H-4′), 3.84 (1H, pt, *J* = 9.5, 9.3 Hz, H-2′ or H-3′ or H-4′), 3.79 (1H, dd, *J* = 11.9, 5.2 Hz, H-6′a), 3.64 (1H, dd, *J* = 11.9, 1.9 Hz, H-6′b), 3.52-3.49 (1H, m, H-5′); ^13^C NMR (100 MHz, CDCl_3_) δ (ppm): 168.8, 163.3 (C-2, C-4), 160.5 (C-6), 138.3, 138.1, 137.7, 136.9, 129.0–127.9 (aromatics), 114.8 (*C*N), 91.0 (C-5), 87.0, 83.2, 82.2, 79.0, 77.5 (C-1′–C-5′), 76.2, 75.5, 75.0, 73.8 (4 × Ph*C*H_2_), 67.8 (C-6′). ESI-MS positive mode (*m/z*): Calcd for C_39_H_39_N_4_O_5_^+^ [M + H]^+^ 643.3. Found: 643.5.

*Ethyl 4-amino-2-(2′,3′,4′,6′-tetra-*O*-benzyl-β-D-glucopyranosyl)-pyrimidine-5-carboxylate* (**10b**) and *2-(2′,3′,4′,6′-tetra-*O*-benzyl-β-D-glucopyranosyl)-6-oxo-1,6-dihydropyrimidine-5-carbonitrile* (**10c**). The title compounds were prepared from compound **1** (400 mg, 0.66 mmol) and ethyl 2-cyano-3-ethoxyacrylate **4** (224 mg, 1.33 mmol) according to general procedure 1. Reaction time: 1 h. Purification by column chromatography (EtOAc-hexane = 1:3) yielded **10b** as the first and **10c** as the second fraction. **10b:** Yield: 167 mg (37%), colourless syrup. R_f_ = 0.25 (EtOAc-hexane = 1:2); [α]_D_ = +54 (c 0.20, CH_2_Cl_2_); ^1^H NMR (400 MHz, CDCl_3_) δ (ppm): 8.81 (1H, s, H-6), 7.84 (1H, br s, NH_2_), 7.31–6.97 (20H, m, aromatics), 6.38 (1H, br s, NH_2_), 4.93, 4.89 (2 × 1H, 2d, *J* = 11.2 Hz in each, PhCH_2_), 4.84, 4.57 (2 × 1H, 2d, *J* = 10.7 Hz in each, PhCH_2_), 4.60, 4.27 (2 × 1H, 2d, *J* = 11.4 Hz in each, PhCH_2_), 4.60, 4.27 (2 × 1H, 2d, *J* = 12.2 Hz in each, PhCH_2_), 4.36 (2H, q, i = 7.2 Hz, CH_2_CH_3_), 4.36 (1H, d, *J* = 9.6 Hz, H-1′), 4.03 (1H, pt, *J* = 9.6, 9.0 Hz, H-2′), 3.84 (1H, pt, *J* = 9.2, 9.0 Hz, H-3′), 3.76–3.3.71 (3H, m, H-4′, H-6′a, H-6′b), 3.65 (1H, ddd, *J* = 9.5, 4.5, 2.2 Hz, H-5′), 1.40 (3H, t, *J* = 7.2 Hz, CH_2_CH_3_); ^13^C NMR (100 MHz, CDCl_3_) δ (ppm): 168.9, 166.0, 162.8 (C-2, C-4, COOEt), 159.6 (C-6), 138.8, 138.2, 138.2, 138.1, 128.5–127.5 (aromatics), 104.3 (C-5), 87.1, 82.9, 81.3, 79.8, 77.3 (C-1′–C-5′), 75.7, 75.2, 74.8, 73.5 (4 × PhCH_2_), 69.1 (C-6′), 61.3 (CH_2_CH_3_), 14.4 (CH_2_CH_3_). ESI-MS positive mode (*m*/*z*): Calcd for C_41_H_44_N_3_O_7_^+^ [M + H]^+^ 690.3. Found: 690.5. **10c**: Yield: 128 mg (30%), colourless syrup. R_f_ = 0.23 (EtOAc-hexane = 1:2); [α]_D_ = ‒12 (c 0.22, CH_2_Cl_2_); ^1^H NMR (400 MHz, CDCl_3_) δ (ppm): 12.52 (1H, br s, NH), 8.15 (1H, s, H-4), 7.33-7.09 (20H, m, aromatics), 4.91, 4.88 (2 × 1H, 2d, *J* = 11.3 Hz in each, PhCH_2_), 4.86, 4.60 (2 × 1H, 2d, *J* = 10.8 Hz in each, PhCH_2_), 4.71, 4.46 (2 × 1H, 2d, *J* = 11.5 Hz in each, PhCH_2_), 4.54, 4.48 (2 × 1H, 2d, *J* = 12.0 Hz in each, PhCH_2_), 4.37 (1H, d, *J* = 9.5 Hz, H-1′), 3.86-3.70 (6H, m, H-2′–H-6′a,b); ^13^C NMR (100 MHz, CDCl_3_) δ (ppm): 162.9, 160.0 (C-2, C-6), 161.1 (C-4), 138.1, 137.9, 137.6, 137.1, 128.7–127.9 (aromatics), 113.3 (CN), 103.2 (C-5), 85.8, 79.2, 78.9, 78.2, 77.7 (C-1′–C-5′), 75.6, 75.2, 74.6, 73.4 (4 × PhCH_2_), 69.0 (C-6′). ESI-MS positive mode (*m*/*z*): Calcd for C_39_H_38_N_3_O_6_^+^ [M + H]^+^ 644.3. Found: 644.5.

*Ethyl 2-(2′,3′,4′,6′-tetra-*O*-benzyl-β-D-glucopyranosyl)-6-oxo-1,6-dihydropyrimidine-5-carboxylate* (**10d**). Prepared from compound **1** (400 mg, 0.66 mmol) and diethyl 2-(ethoxymethylene)malonate **5** (265 μL, 1.33 mmol) according to general procedure 1. Reaction time: 1 h. Purified by column chromatography (EtOAc-hexane 1:1) to give 367 mg (80%) colourless syrup. R_f_ = 0.21 (EtOAc-hexane = 1:1); [α]_D_ = +9 (c 0.50, CH_2_Cl_2_); ^1^H NMR (400 MHz, CDCl_3_) δ (ppm): 11.35 (1H, br s, NH), 8.55 (1H, s, H-4), 7.32–7.11 (20H, m, aromatics), 4.88, 4.84 (2 × 1H, 2d, *J* = 11.2 Hz in each, Ph*CH_2_*), 4.82, 4.55 (2 × 1H, 2d, *J* = 10.9 Hz in each, Ph*CH_2_*), 4.66, 4.45 (2 × 1H, 2d, *J* = 11.4 Hz in each, Ph*CH_2_*), 4.55, 4.48 (2 × 1H, 2d, *J* = 12.1 Hz in each, Ph*CH_2_*), 4.37 (2H, q, *J* = 7.2 Hz, *CH_2_*CH_3_), 4.37 (1H, d, *J* = 9.5 Hz, H-1′), 3.86–3.65 (6H, m, H-2′–H-6′a,b), 1.38 (3H, t, *J* = 7.2 Hz, CH_2_*CH_3_*); ^13^C NMR (90 MHz, CDCl_3_) δ (ppm): 163.2, 162.6, 160.0 (C-2, C-6, *C*OOEt), 159.2 (C-4), 138.2, 138.0, 137.6, 137.2, 128.5–127.8 (aromatics), 116.7 (C-5), 86.1, 79.4, 78.9, 78.4, 77.4 (C-1′–C-5′), 75.5, 75.0, 74.6, 73.4 (4 × Ph*C*H_2_), 68.8 (C-6′), 61.3 (*C*H_2_CH_3_), 14.4 (CH_2_*C*H_3_). ESI-MS positive mode (*m/z*): Calcd for C_41_H_43_N_2_O_8_^+^ [M + H]^+^ 691.3. Found: 691.4.

*4-Amino-2-(2′,3′,4′,6′-tetra-*O*-benzyl-β-D-glucopyranosyl)-6-phenylpyrimidine-5-carbonitrile* (**10e**). Prepared from compound **1** (400 mg, 0.66 mmol) and 2-benzylidenemalononitrile **6** (204 mg, 1.33 mmol) according to general procedure 1. Reaction time: 1 h. The title compound precipitated from the reaction mixture was a pale yellow amorphous solid. Yield: 373 mg (78%). R_f_ = 0.41 (EtOAc-hexane = 2:3); [α]_D_ = ‒12 (c 0.27, CH_2_Cl_2_); ^1^H NMR (400 MHz, CDCl_3_) δ (ppm): 9.00 (2H, br s, NH_2_), 7.96–6.97 (25H, m, aromatics), 4.96–4.69 (6H, m, Ph*CH_2_*), 4.73, 4.27 (2 × 1H, 2d, *J* = 11.3 Hz in each, Ph*CH_2_*), 4.50 (1H, d, *J* = 9.6 Hz, H-1′), 4.03 (1H, pt, *J* = 9.5, 9.2 Hz, H-4′), 3.98 (1H, pt, *J* = 9.6, 9.1 Hz, H-2′), 3.85 (1H, pt, *J* = 9.2, 9.1 Hz, H-3′), 3.82 (1H, dd, *J* = 11.8, 3.8 Hz, H-6′a), 3.66 (1H, dd, *J* = 11.8, 1.9 Hz, H-6′b), 3.52 (1H, ddd, *J* = 9.5, 3.8, 1.9 Hz, H-5′); ^13^C NMR (100 MHz, CDCl_3_) δ (ppm): 168.3, 167.9, 165.4 (C-2, C-4, C-6), 138.5, 138.2, 137.8, 137.1, 136.0, 131.4, 129.0–127.9 (aromatics), 116.0 (*C*N), 87.4 (C-5), 87.1, 83.5, 82.5, 79.0, 77.7 (C-1′–C-5′), 76.2, 75.6, 75.3, 73.9 (4 × Ph*C*H_2_), 67.9 (C-6′). ESI-MS positive mode (*m/z*): Calcd for C_45_H_43_N_4_O_5_^+^ [M + H]^+^ 719.3. Found: 791.6.

*2-(2′,3′,4′,6′-Tetra-*O*-benzyl-β-D-glucopyranosyl)-4-phenyl-6-oxo-1,6-dihydropyrimidine-5-carbonitrile* (**10f**). Prepared from compound **1** (400 mg, 0.66 mmol) and ethyl 2-cyano-3-phenylacrylate **7** (267 mg, 1.33 mmol) according to general procedure 1. Reaction time: 1 h. Purified by column chromatography (EtOAc-hexane = 2:3) to give 334 mg (70%) colourless syrup. R_f_ = 0.51 (EtOAc-hexane = 1:1); [α]_D_ = +11 (c 0.25, CH_2_Cl_2_); ^1^H NMR (400 MHz, CDCl_3_) δ (ppm): 8.07–7.02 (25H, m, aromaics), 4.98, 4.93 (2 × 1H, 2d, *J* = 11.2 Hz in each, Ph*CH_2_*), 4.93, 4.72 (2 × 1H, 2d, *J* = 10.7 Hz in each, Ph*CH_2_*), 4.77, 4.47 (2 × 1H, 2d, *J* = 11.3 Hz in each, Ph*CH_2_*), 4.61, 4.53 (2 × 1H, 2d, *J* = 12.3 Hz in each, Ph*CH_2_*), 4.52 (1H, d, *J* = 9.5 Hz, H-1′), 3.97-3.84 (6H, m, H-2′–H-6′a,b); ^13^C NMR (100 MHz, CDCl_3_) δ (ppm): 168.7, 162.8, 160.6 (C-2, C-4, C-6), 138.2, 138.1, 137.8, 137.1, 134.6, 132.3, 129.3-127.6 (aromatics), 114.8 (*C*N), 97.7 (C-5), 86.1, 79.7, 79.2, 78.7, 78.1 (C-1′–C-5′), 75.6, 75.4, 74.7, 73.3 (4 × Ph*C*H_2_), 69.4 (C-6′). ESI-MS positive mode (*m/z*): Calcd for C_45_H_42_N_3_O_6_^+^ [M + H]^+^ 720.3. Found: 720.6.

*Methyl 2-(2′,3′,4′,6′-tetra-O-benzyl-β-D-glucopyranosyl)-4-phenyl-6-oxo-1,6-dihydropyrimidine-5-carboxylate* (**10g**). Prepared from compound **12g** (300 mg, 0.40 mmol) and DDQ (90 mg, 0.40 mmol) according to general procedure 3. Purified by column chromatography (EtOAc-hexane = 2:3) to give 177 mg (59%) pale yellow syrup. R_f_ = 0.48 (EtOAc-hexane = 1:1); [α]_D_ = +10 (c 0.40, CH_2_Cl_2_); ^1^H NMR (360 MHz, CDCl_3_) δ (ppm): 12.40 (1H, br s, NH), 7.65–7.01 (25H, m, aromatics), 4.93, 4.90 (2 × 1H, 2d, *J* = 11.5 Hz, Ph*CH_2_*), 4.88, 4.64 (2 × 1H, 2d, *J* = 10.8 Hz, Ph*CH_2_*), 4.73, 4.47 (2 × 1H, 2d, *J* = 11.0 Hz, Ph*CH_2_*), 4.58, 4.54 (2 × 1H, 2d, *J* = 12.1 Hz, Ph*CH_2_*), 4.42 (1H, d, *J* = 9.4 Hz, H-1′), 3.94 (1H, pt, *J* = 9.2, 9.0 Hz, H-2′or H-3′ or H-4′), 3.86–3.72 (5H, m, H-2′ and/or H-3′ and/or H-4′, H-5′–H-6′), 3.64 (3H, s, OCH_3_); ^13^C NMR (90 MHz, CDCl_3_) δ (ppm): 166.0, 161.3 (2), 157.9 (C-2, C-4, C-6, COOMe), 138.3, 138.0, 137.9, 137.2, 136.8, 130.5, 128.5-127.8 (aromatics), 118.4 (C-5), 86.3, 79.2, 79.2, 78.8, 77.8 (C-1′–C-5′), 75.7, 75.3, 74.7, 73.4 (4 × Ph*C*H_2_), 69.0 (C-6′), 52.6 (O*C*H_3_). ESI-MS positive mode (*m/z*): Calcd for C_46_H_45_N_2_O_8_^+^ [M + H]^+^ 753.3. Found: 753.6.

*Ethyl 2-(2′,3′,4′,6′-tetra-*O*-benzyl-β-D-glucopyranosyl)-4-phenyl-6-oxo-1,6-dihydropyrimidine-5-carboxylate* (**10h**). Prepared from compound **12h** (300 mg, 0.40 mmol) and DDQ (90 mg, 0.40 mmol) according to general procedure 3. Purified by column chromatography (EtOAc-hexane = 2:3) to give 159 mg (53%) pale yellow syrup. R_f_ = 0.50 (EtOAc-hexane = 1 : 1); [α]_D_ = +62 (c 0.23, CH_2_Cl_2_); ^1^H NMR (360 MHz, CDCl_3_) δ (ppm): 12.67 (1H, br s, NH), 7.66–7.02 (25H, m, aromatics), 4.94, 4.91 (2 × 1H, 2d, *J* = 11.2 Hz in each, Ph*CH_2_*), 4.89, 4.64 (2 × 1H, 2d, *J* = 10.8 Hz in each, Ph*CH_2_*), 4.74, 4.49 (2 × 1H, 2d, *J* = 11.2 Hz in each, Ph*CH_2_*), 4.58, 4.52 (2 × 1H, 2d, *J* = 12.2 Hz in each, Ph*CH_2_*), 4.43 (1H, d, *J* = 9.5 Hz, H-1′), 4.14 (2H, q, *J* = 7.1 Hz, *CH_2_*CH_3_), 3.97 (1H, pt, *J* = 9.2, 9.0 Hz, H-2′ or H-3′ or H-4′), 3.87–3.70 (5H, m, H-2′ and/or H-3′ and/or H-4′, H-5′–H-6′a,b), 1.00 (3H, t, *J* = 7.1 Hz, CH_2_*CH_3_*); ^13^C NMR (90 MHz, CDCl_3_) δ (ppm): 165.3, 161.4, 161.3, 157.9 (C-2, C-4, C-6, COOEt), 138.4, 138.1, 137.8, 137.2, 136.9, 130.3, 128.5-127.7 (aromatics), 118.7 (C-5), 86.4, 79.2 (2), 78.9, 77.8 (C-1′–C-5′), 75.7, 75.2, 74.7, 73.4 (4 × Ph*C*H_2_), 69.0 (C-6′), 61.6 (*C*H_2_CH_3_), 13.8 (CH_2_*C*H_3_). ESI-MS positive mode (*m/z*): Calcd for C_47_H_47_N_2_O_8_^+^ [M + H]^+^ 767.3. Found: 767.6.

*4-Amino-2-(β-D-glucopyranosyl)-pyrimidine-5-carbonitrile* (**11a**). Prepared from compound **2** (100 mg, 0.41 mmol) and 2-(ethoxymethylene)malononitrile **3** (101 mg, 0.82 mmol) according to general procedure 1. Reaction time: 30 min. Purified by column chromatography (CHCl_3_-MeOH = 5:1) to give 85 mg (73%) pale yellow syrup. R_f_ = 0.31 (CHCl_3_-MeOH = 3:1); [α]_D_ = +42 (c 0.16, MeOH); ^1^H NMR (400 MHz, CD_3_OD) δ (ppm): 8.56 (1H, s, H-6), 4.18 (1H, d, *J* = 9.5 Hz, H-1′), 3.85 (1H, dd, *J* = 12.2, 1.9 Hz, H-6′a), 3.70 (1H, dd, *J* = 12.2, 4.9 Hz, H-6′b), 3.67 (1H, pt, *J* = 9.5, 9.0 Hz, H-2′), 3.50 (1H, pt, *J* = 9.1, 9.0 Hz, H-3′), 3.44 (1H, pt, *J* = 9.4, 9.1 Hz, H-4′), 3.39 (1H, ddd, *J* = 9.4, 4.9, 1.9 Hz, H-5′); ^13^C NMR (100 MHz, CD_3_OD) δ (ppm): 170.2, 164.4 (C-2, C-4), 161.8 (C-6), 115.4 (*C*N), 90.8 (C-5), 83.5, 82.3, 79.2, 74.4, 71.1 (C-1′–C-5′), 62.7 (C-6′). ESI-HRMS positive mode (*m/z*): calcd for C_11_H_15_N_4_O_5_^+^ [M + H]^+^ 283.1037; C_11_H_14_N_4_NaO_5_^+^ [M + Na]^+^ 305.0856. Found: [M + H]^+^ 283.1034; [M + Na]^+^ 305.0852.

*Ethyl 4-amino-2-(β-D-glucopyranosyl)-pirimidine-5-carboxylate* (**11b**) and *2-(β-D-glucopyranosyl)-6-oxo-1,6-dihydropyrimidine-5-carbonitrile* (**11c**). Method A: The Pd-catalyst (35 mg, 20% Pd(OH)_2_/C) was suspended in an anhydrous EtOAc-EtOH solvent mixture (10 mL in 1:5 ratio) under Ar and the suspension was saturatated with H_2_ (3×). To this heterogenous mixture, a solution of compound **10b** (70 mg, 0.10 mmol) in EtOAc (1 mL) and one drop of ccHCl were added. The reaction mixture was stirred under H_2_ at rt for two days, and the transformation was monitored by TLC (EtOAc-hexane 1:1 and CHCl_3_-MeOH 7:3). After complete conversion of the starting material, the mixture was neutralized with NaHCO_3_. The catalyst and insoluble inorganic salts were filtered off through a pad of Celite and washed three times with MeOH (3 × 3 mL). The resulting combined solution was concentrated under diminished pressure and the residue was purified by column chromatography (CHCl_3_-MeOH = 5:1). Yield of compound **11b**: 17 mg (51%) colourless syrup. R_f_ = 0.55 (CHCl_3_-MeOH = 7:3); [α]_D_ = ‒62 (c 0.15, MeOH); ^1^H NMR (400 MHz, D_2_O) δ (ppm): 8.82 (1H, s, H-6), 4.37 (2H, q, *J* = 7.1 Hz, CH_2_CH_3_), 4.28 (1H, d, *J* = 9.4 Hz, H-1′), 3.93 (1H, dd, *J* = 12.2, 1.9 Hz, H-6′a), 3.81 (1H, dd, *J* = 12.2, 4.5 Hz, H-6′b), 3.73 (1H, pt, *J* = 9.4, 9.0 Hz, H-2′), 3.69–3.54 (3H, m, H-3′, H-4′, H-5′), 1.38 (3H, t, *J* = 7.1 Hz, CH_2_CH_3_); ^13^C NMR (90 MHz, CD_3_OD) δ (ppm): 170.4, 166.9, 164.0, 159.7 (C-2, C-4, C-6, COOEt), 105.2 (C-5), 83.0, 82.3, 79.3, 74.5, 71.2 (C-1′–C-5′), 62.9 (C-6′), 62.4 (CH_2_CH_3_), 14.5 (CH_2_CH_3_). ESI-HRMS positive mode (*m*/*z*): calcd for C_13_H_20_N_3_O_7_^+^ [M + H]^+^ 330.1296; C_13_H_19_N_3_NaO_7_^+^ [M + Na]^+^ 352.1115. Found: [M + H]^+^ 330.1294; [M + Na]^+^ 352.1114. Method B: The title compounds **11b** and **11c** were prepared from compound **2** (200 mg, 0.82 mmol) and ethyl 2-cyano-3-ethoxyacrylate **4** (279 mg, 1.65 mmol) according to general procedure 1. Reaction time: 1 h. Purification by column chromatography (CHCl_3_-MeOH = 5:1 → 3:1) yielded **11b** (55 mg, 20 %) as the first and **11c** 105 mg (45%) as the second fraction. Compound **11c**: colourless syrup. R_f_ = 0.39 (CHCl_3_-MeOH 1:1); [α]_D_ = ‒66 (c 0.16, MeOH); ^1^H NMR (400 MHz, CD_3_OD) δ (ppm): 8.33 (1H, s, H-4), 4.11 (1H, d, *J* = 9.4 Hz, H-1′), 3.88 (1H, dd, *J* = 12.0, 1.8 Hz, H-6′a), 3.71 (1H, dd, *J* = 12.0, 4.8 Hz, H-6′b), 3.58 (1H, pt, *J* = 9.3, 9.2 Hz, H-2′ or H-3′ or H-4′), 3.53–3.41 (3H, m, H-2′ and/or H-3′ and/or H-4′, H-5′); ^13^C NMR (100 MHz, CD_3_OD) δ (ppm): 173.1, 171.1 (C-2, C-6), 161.4 (C-4), 118.1 (CN), 97.6 (C-5), 81.8, 81.4, 79.1, 74.6, 71.0 (C-1′–C-5′), 62.5 (C-6′). ESI-HRMS positive mode (*m*/*z*): Calcd. for C_11_H_13_NaN_3_O_6_^+^ [M + Na]^+^ 306.0697. Found: 306.0696.

*Ethyl 2-(β-D-glucopyranosyl)-6-oxo-1,6-dihydropyrimidine-5-carboxylate* (**11d**). Method A: The Pd-catalyst (150 mg, 20% Pd(OH)_2_/C) was suspended in an anhydrous EtOAc-EtOH solvent mixture (30 mL in 1:5 ratio) under Ar. This degased suspension was saturatated with H_2_ (3×). To this heterogenous mixture, a solution of compound **10d** (335 mg, 0.48 mmol) in EtOAc (3 mL) and three drops of ccHCl were added. The reaction mixture was stirred under H_2_ at rt for two days, and the transformation was monitored by TLC (EtOAc-hexane 1:1 and CHCl_3_-MeOH 7:3). After the complete conversion of the starting material, the mixture was neutralized with NaHCO_3_. The catalyst and the insoluble inorganic salts were filtered off through a pad of Celite and washed three times with MeOH (3 × 10 mL). The resulting solution was concentrated under reduced pressure and the residue was purified by column chromatography (CHCl_3_-MeOH = 3:1). Yield: 107 mg (67%), colourless syrup. Method B: Prepared from compound **2** (100 mg, 0.41 mmol) and diethyl 2-(ethoxymethylene)malonate **5** (165 μL, 0.82 mmol) according to general procedure 1. Reaction time: 1 h. Purified by column chromatography (CHCl_3_-MeOH = 3:1) to give 70 mg (51%) colourless syrup. R_f_ = 0.38 (CHCl_3_-MeOH = 1:1); [α]_D_ = +75 (c 0.15, MeOH); ^1^H NMR (360 MHz, D_2_O) δ (ppm): 8.70 (1H, s, H-4), 4.39–4.32 (3H, m, H-1′, *CH_2_*CH_3_), 3.94 (1H, dd, *J* = 12.2, 2.4 Hz, H-6′a), 3.82 (1H, dd, *J* = 12.2, 4.0 Hz, H-6′b), 3.72–3.60 (4H, m, H-2′–H-5′), 1.36 (3H, t, *J* = 6.9 Hz, CH_2_*CH_3_*); ^13^C NMR (90 MHz, D_2_O) δ (ppm): 167.2, 166.7, 164.9, 157.3 (C-2, C-4, C-6, COOEt), 114.2 (C-5), 80.4, 79.7, 77.3, 73.0, 69.7 (C-1′–C-5′), 62.8 (C-6′), 61.2 (*C*H_2_CH_3_), 14.0 (CH_2_*C*H_3_). ESI-HRMS positive mode (*m/z*): calcd for C_13_H_19_N_2_O_8_^+^ [M + H]^+^ 331.1136; C_13_H_18_N_2_NaO_8_^+^ [M + Na]^+^ 353.0955. Found: [M + H]^+^ 331.1140; [M + Na]^+^ 353.0953.

*4-Amino-2-(β-D-glucopyranosyl)-6-phenyl-pirimidine-5-carbonitrile* (**11e**). Prepared from compound **2** (50 mg, 0.21 mmol) and 2-benzylidenemalononitrile **6** (64 mg, 0.41 mmol) according to general procedure 1. Reaction time: 1 h. Purified by column chromatography (CHCl_3_-MeOH = 3:1) to give 63 mg (85%) pale yellow syrup. R_f_ = 0.49 (CHCl_3_-MeOH 7:3); [α]_D_ = +34 (c 0.17, MeOH); ^1^H NMR (400 MHz, CD_3_OD) δ (ppm): 7.89-7.87 (2H, d, *J* = 7.9 Hz, Ph), 7.57–7.50 (3H, m, Ph ), 4.29 (1H, d, *J* = 9.6 Hz, H-1′), 3.86 (1H, dd, *J* = 12.1, 2.1 Hz, H-6′a), 3.81 (1H, pt, *J* = 9.5, 9.3 Hz, H-2′), 3.77 (1H, dd, *J* = 12.1, 4.7 Hz, H-6′b), 3.59–3.52 (2H, m, H-3′, H-4′), 3.45–3.42 (1H, m, H-5′); ^13^C NMR (100 MHz, CD_3_OD) δ (ppm): 170.4, 169.5, 166.2 (C-2, C-4, C-6), 137.5, 132.2, 129.9, 129.8, 129.6 (2) (Ph), 116.4 (*C*N), 90.8 (C-5), 87.8, 83.8, 82.2, 79.0, 70.9 (C-1′–C-5′), 62.4 (C-6′). ESI-HRMS positive mode (*m/z*): Calcd for C_17_H_19_N_4_O_5_^+^ [M + H]^+^ 359.1350; C_13_H_18_N_2_NaO_8_^+^ [M + Na]^+^ 381.1169. Found: [M + H]^+^ 359.1350; [M + Na]^+^ 381.1169.

*2-(β-D-Glucopyranosyl)-4-phenyl-6-oxo-1,6-dihydropyrimidine-5-carbonitrile* (**11f**). Prepared from compound **2** (50 mg, 0.21 mmol) and ethyl 2-cyano-3-phenylacrylate **7** (83 mg, 0.41 mmol) according to general procedure 1. Reaction time: 1 h. Purified by column chromarography (CHCl_3_-MeOH = 7:3) to give 30 mg (41%) colourless syrup. R_f_ = 0.21 (CHCl_3_-MeOH 7:3); [α]_D_ = +23 (c 0.16, MeOH); ^1^H NMR (360 MHz, D_2_O) δ (ppm): 7.81 (2H, d, *J* = 6.9 Hz, Ph), 7.64–7.58 (3H, m, Ph), 4.31 (1H, d, *J* = 9.5 Hz, H-1′), 3.91–3.78 (3H, m, H-2′, H-6′a, H-6′b), 3.69–3.60 (3H, m, H-3′–H-5′); ^13^C NMR (90 MHz, D_2_O) δ (ppm): 173.2, 171.1, 167.5 (C-2, C-4, C-6), 136.3, 131.7, 129.3 (2), 129.0 (2) (Ph), 118.7 (*C*N), 95.1 (C-5), 81.9, 80.4, 77.5, 73.3, 69.7 (C-1′–C-5′), 61.1 (C-6′). ESI-HRMS positive mode (*m/z*): Calcd for C_17_H_18_N_3_O_6_^+^ [M + H]^+^ 360.1190; C_17_H_17_N_3_NaO_6_^+^ [M + Na]^+^ 382.1010. Found: [M + H]^+^ 360.1190; [M + Na]^+^ 382.1009.

*Methyl 2-(β-D-glucopyranosyl)-4-phenyl-6-oxo-1,6-dihydropyrimidine-5-carboxylate* (**11g**). The Pd-catalyst (50 mg, 20% Pd(OH)_2_/C) was suspended in anhydrous EtOH (10 mL) under Ar. This degased suspension was saturatated with H_2_ (3×). To this heterogenous mixture, a solution of compound **10g** (200 mg, 0.27 mmol) in anhydrous EtOAc (2 mL) was added. The reaction mixture was heated at reflux temperature under H_2_ atmosphere until the TLC indicated (EtOAc-hexane 1:1 and CHCl_3_-MeOH 4:1) the complete conversion of the starting material (6 h). After the completion of the reaction, the catalyst was filtered off through a pad of Celite, and washed with MeOH (3 × 5 mL). The combined organic solution was concentrated under reduced pressure and the crude product was purified by column chromatography (CHCl_3_-MeOH = 8:1). Yield: 73 mg (70%), colourless syrup. R_f_ = 0.43 (CHCl_3_-MeOH = 7:1); [α]_D_ = +37 (c 0.18, MeOH); ^1^H NMR (400 MHz, CD_3_OD) δ (ppm): 7.63–7.43 (5H, m, Ph), 4.28 (1H, d, *J* = 9.5 Hz, H-1′), 3.90 (1H, dd, *J* = 12.0, 2.0 Hz, H-6′a), 3.79 (1H, dd, *J* = 12.0, 4.3 Hz, H-6′b), 3.68 (3H, s, OCH_3_), 3.65 (1H, pt, *J* = 9.5, 9.2 Hz, H-2′), 3.55–3.43 (3H, m, H-3′, H-4′, H-5′); ^13^C NMR (100 MHz, CD_3_OD) δ (ppm): 167.7, 162.8, 162.2, 161.2 (C-2, C-4, C-6, COOMe), 137.9, 131.6, 129.6 (2), 129.2 (2) (Ph), 119.4 (C-5), 82.1, 80.0, 78.8, 74.0, 70.5 (C-1′–C-5′), 62.2 (C-6′), 53.0 (O*C*H_3_). ESI-HRMS positive mode (*m/z*): Calcd for C_18_H_21_N_2_O_8_^+^ [M + H]^+^ 393.1292; C_18_H_20_N_2_NaO_8_^+^ [M + Na]^+^ 415.1112. Found: [M + H]^+^ 393.1292; [M + Na]^+^ 415.1111.

*Ethyl 2-(β-D-glucopyranosyl)-4-phenyl-6-oxo-1,6-dihydropyrimidine-5-carboxylate* (**11h**). The Pd-catalyst (50 mg, 20% Pd(OH)_2_/C) was suspended in anhydrous EtOH (10 mL) under Ar, and the suspension was saturatated with H_2_ (3×). To this heterogenous mixture, a solution of compound **11h** (200 mg, 0.26 mmol) in anhydrous EtOAc (2 mL) was added. The reaction mixture was heated at reflux temperature under H_2_ atmosphere until the TLC indicated (EtOAc-hexane 1:1 and CHCl_3_-MeOH 4:1) the complete conversion of the starting material (6 h). After completion of the reaction, the catalyst was filtered off through a pad of Celite, and washed with MeOH (3 × 5 mL). The combined organic solution was concentrated under reduced pressure and the crude product was purified by column chromatography (CHCl_3_-MeOH = 8:1). Yield: 61 mg (58%), colourless syrup. R_f_ = 0.43 (CHCl_3_-MeOH = 7:1); [α]_D_ = +62 (c 0.11, MeOH); ^1^H NMR (400 MHz, CD_3_OD) δ (ppm): 7.63–7.43 (5H, m, Ph), 4.28 (1H, d, *J* = 9.5 Hz, H-1′), 4.16 (2H, q, *J* = 7.1 Hz, *CH_2_*CH_3_), 3.90 (1H, dd, *J* = 11.9, 2.0 Hz, H-6′a), 3.79 (1H, dd, *J* = 11.9, 4.3 Hz, H-6′b), 3.64 (1H, pt, *J* = 9.5, 9.1 Hz, H-2′), 3.54-3.46 (3H, m, H-3′, H-4′, H-5′), 1.08 (3H, t, *J* = 7.1 Hz, CH_2_*CH_3_*); ^13^C NMR (90 MHz, CD_3_OD) δ (ppm): 167.2, 162.9, 162.2, 161.2 (C-2, C-4, C-6, COOEt), 138.1, 131.5, 129.5 (2), 129.3 (2) (Ph), 119.7 (C-5), 82.1, 80.1, 78.8, 74.0, 70.5 (C-1′–C-5′), 62.8 (C-6′), 62.2 (*C*H_2_CH_3_), 14.0 (CH_2_*C*H_3_). ESI-HRMS positive mode (*m/z*): calcd for C_19_H_23_N_2_O_8_^+^ [M + H]^+^ 407.1449; C_19_H_22_N_2_NaO_8_^+^ [M + Na]^+^ 429.1268. Found: [M + H]^+^ 407.1445; [M + Na]^+^ 429.1262.

*Methyl 2-(2′,3′,4′,6′-tetra-*O*-benzyl-β-D-glucopyranosyl)-4-phenyl-6-oxo-1,4,5,6-tetrahydropyrimi-dine-5-carboxylate* (**12g**). Prepared from compound **1** (400 mg, 0.66 mmol) and dimethyl benzylidenemalonate **8** (292 mg, 1.33 mmol) according to general procedure 2. Purified by column chromatography (EtOAc-hexane = 2:3) to give 451 mg (90%) colourless syrup. R_f_ = 0.46 (EtOAc-hexane = 2:3). ^1^H NMR (400 MHz, CDCl_3_) δ (ppm): 8.74, 8.67 (br s, 2 × NH), 7.33–7.10 (m, aromatics), 5.00 (d, *J* = 13.5 Hz, H-4 or H-5), 4.94 (d, *J* = 11.6 Hz, H-4 or H-5), 4.87–4.48 (m, Ph*CH_2_*), 4.10, 4.05 (2d, *J* = 9.1 Hz in each, 2 × H-1′), 3.79–3.55 (m, 2 × [H-2′–H-6′a,b]), 3.61, 3.55 (2s, 2 × OMe), 3.49 (d, *J* = 11.5 Hz, H-4 or H-5), 3.22 (d, *J* = 13.8 Hz, H-4 or H-5); ^13^C NMR (100 MHz, CDCl_3_) δ (ppm): 168.3, 168.1, 166.2 (2) (2 × [C-6, COOMe]), 150.7, 150.5 (2 × C-2), 139.9 (2), 138.4, 138.2, 138.0, 137.9, 137.9, 137.8, 137.7 (2), 128.8-127.2 (aromatics), 86.3 (2), 79.2, 79.1, 79.1, 78.9, 78.7 (2), 77.4 (2) (2 × [C-1′–C-5′]), 75.7 (2), 75.2 (2), 74.8 (2), 73.6, 73.6 (8 × Ph*C*H_2_), 68.8, 68.6 (2 × C-6′), 61.5, 61.4, 53.8, 53.7, 52.7, 52.7 (2 × [C-4, C-5, O*C*H_3_]). ESI-HRMS positive mode (*m/z*): Calcd for C_46_H_47_N_2_O_8_^+^ [M + H]^+^ = 755.3. Found: 755.5. 

*Ethyl 2-(2′,3′,4′,6′-tetra-*O*-benzyl-β-D-glucopyranosyl)-4-phenyl-6-oxo-1,4,5,6-tetrahydropyrimidine-5-carboxylate* (**12h**). Prepared from compound **2** (400 mg, 0.66 mmol) and diethyl benzylidinemalonate **9** (329 mg, 1.33 mmol) according to general procedure 2. Purified by column chromatography (EtOAc-hexane = 2:3) to give 413 mg (81%) colourless syrup. R_f_ = 0.46 (EtOAc-hexane = 2:3). ^1^H NMR (400 MHz, CDCl_3_) δ (ppm): 8.68, 8.60 (br s, 2 × NH), 7.36–7.11 (m, aromatics), 4.99 (d, *J* = 13.3 Hz, H-4 or H-5), 4.92 (d, *J* = 11.8 Hz, H-4 or H-5), 4.90-4.49 (m, Ph*CH_2_*), 4.08, 4.07 (2q, *J* = 7.1 Hz in each, 2 × *CH_2_*CH_3_), 4.05, 4.01 (2d, *J* = 9.1 Hz in each, 2 × H-1′), 3.79–3.53 (m, 2 × [H-2′–H-6′]), 3.47 (d, *J* = 11.8 Hz, H-4 or H-5), 3.20 (d, *J* = 13.4 Hz, H-4 or H-5), 1.08, 1.06 (2t, *J* = 7.1 Hz in each, 2 × CH_2_*CH_3_*); ^13^C NMR (100 MHz, CDCl_3_) δ (ppm): 167.8, 167.6, 166.3, 166.2 (2 × C-6, 2 × COOEt), 150.7, 150.5 (2 × C-2), 139.9, 139.9, 138.4, 138.2, 138.0, 137.9, 137.9, 137.8, 137.7, 137.7, 128.7–127.3 (aromatics), 86.3 (2), 79.2, 79.1, 79.1, 78.9, 78.7, 78.7, 77.5, 77.4 (2 × [C-1′–C-5′]), 75.7 (2), 75.2 (2), 74.8 (2), 73.6, 73.6, (8 × Ph*C*H_2_), 68.8, 68.6 (2 × C-6′), 61.8, 61.7 (2 × *C*H_2_CH_3_), 61.6, 61.5, 53.8 (2) (2 × C-4, 2 × C-5), 14.0, 14.0 (2 × CH_2_*C*H_3_). ESI-MS positive mode (*m/z*): Calcd for C_47_H_49_N_2_O_8_^+^ [M + H]^+^ 769.4. Found: 769.6.

*2-Bromo-1,3-bis(dimethylamino)trimethinium perchlorate* (**15**). 1,3-Bis(dimethylamino)trimethinium perchlorate **13** (5 g, 22.06 mmol) and NBS (3.93 g, 22.06 mmol) were stirred in dry CH_2_Cl_2_ at rt for 5 h. The solvent was then removed under diminished pressure and the residue was triturated with cold EtOH (15 mL) and the precipitate was filtered off. The obtained pale yellow solid (yield: 6.67 g, 99%) was used in the next step without further purification. ^1^H NMR (400 MHz, DMSO-d_6_) δ (ppm): 7.95 (2H, s), 3.43 (6H, s), 3.23 (6H, s); ^13^C NMR (100 MHz, DMSO-d_6_) δ (ppm): 161.9, 75.9, 49.4, 39.8.

*2-(2′,3′,4′,6′-Tetra-*O*-benzyl-β-D-glucopyranosyl)-pyrimidine*(**17a**). Prepared from amidine **1** (200 mg, 0.33 mmol) and 1,3-bis(dimethylamino)trimethinium perchlorate **13** (82 mg, 0.36 mmol) according to general procedure 4. Reaction time: 16 h. Purified by column chromatography (EtOAc-hexane = 1:2) to give 120 mg (60 %) white solid. Mp: 87–89 °C; R_f_ = 0.45 (EtOAc-hexane = 1:1); [α]_D_ = +66 (c 0.27, CH_2_Cl_2_); ^1^H NMR (400 MHz, CDCl_3_) δ (ppm): 8.72 (2H, d, *J* = 4.9 Hz, H-4, H-6), 7.34–6.85 (21H, m, aromatics, H-5), 4.94, 4.91 (2 × 1H, 2d, *J* = 11.2 Hz, Ph*CH_2_*), 4.85, 4.57 (2 × 1H, 2d, *J* = 10.8 Hz, Ph*CH_2_*), 4.59, 4.16 (2 × 1H, 2d, *J* = 11.3 Hz, Ph*CH_2_*), 4.57 (1H, d, *J* = 9.6 Hz, H-1′), 4.55, 4.50 (2 × 1H, 2d, *J* = 12.2 Hz, Ph*CH_2_*), 4.15 (1H, pt, *J* = 9.6, 9.2 Hz, H-2′), 3.90 (1H, pt, *J* = 9.2, 9.1 Hz, H-3′), 3.77–3.70 (4H, m, H-4′–H-6′a,b); ^13^C NMR (100 MHz, CDCl_3_) δ (ppm): 166.5 (C-2), 157.3 (C-4, C-6), 138.8, 138.2, 138.1 (2), 128.5-127.5 (aromatics), 120.6 (C-5), 87.2, 83.2, 81.4, 79.9, 78.4 (C-1′–C-5′), 75.7, 75.2, 74.7, 73.5 (4 × Ph*C*H_2_), 69.3 (C-6′). ESI-MS positive mode (*m/z*): Calcd for C_38_H_39_N_2_O_5_^+^ [M + H]^+^ 603.3; Found: [M + H]^+^ 603.5.

*2-(2′,3′,4′,6′-Tetra-*O*-benzyl-β-D-glucopyranosyl)-5-chloropyrimidine* (**17b**). Prepared from amidine **1** (200 mg, 0.33 mmol) and 2-chloro-1,3-bis(dimethylamino)trimethinium hexafluorophosphate **14** (111 mg, 0.36 mmol) according to general procedure 4. Reaction time: 6 h. Purified by column chromatography (EtOAc-hexane = 1:3) to give 205 mg (97%) white solid. Mp: 68–70 °C; R_f_ = 0.40 (EtOAc-hexane = 1 : 3); [α]_D_ = +4 (c 0.25, CH_2_Cl_2_); ^1^H NMR (400 MHz, CDCl_3_) δ (ppm): 8.54 (2H, s, H-4, H-6), 7.36–6.86 (20H, m, aromatics), 4.94 (2H, s, Ph*CH_2_*), 4.85, 4.56 (2 × 1H, 2d, *J* = 10.7 Hz, Ph*CH_2_*), 4.64, 4.26 (2 × 1H, 2d, *J* = 11.6 Hz, Ph*CH_2_*), 4.52 (1H, d, *J* = 9.7 Hz, H-1′), 4.54, 4.49 (2 × 1H, 2d, *J* = 12.4 Hz, Ph*CH_2_*), 4.06 (1H, pt, *J* = 9.7, 9.3 Hz, H-2′), 3.89 (1H, pt, *J* = 9.3, 9.2 Hz, H-3′), 3.75–3.67 (4H, m, H-4′–H-6′a,b); ^13^C NMR (100 MHz, CDCl_3_) δ (ppm): 164.1 (C-2), 155.6 (C-4, C-6), 138.7, 138.2, 138.1, 138.0, 128.6–127.5 (aromatics), 130.8 (C-5), 87.3, 82.3, 80.9, 79.9, 78.4 (C-1′–C-5′), 75.8, 75.2, 74.7, 73.6 (4 × Ph*C*H_2_), 69.2 (C-6′). ESI-MS positive mode (*m/z*): Calcd for C_38_H_38_ClN_2_O_5_^+^ [M + H]^+^ 637.2464; C_38_H_37_ClN_2_NaO_5_^+^ [M + Na]^+^ 659.2283. Found: [M + H]^+^ 637.2464; [M + Na]^+^ 659.2284.

*2-(2′,3′,4′,6′-Tetra-O-benzyl-β-D-glucopyranosyl)-5-bromopyrimidine* (**17c**). Prepared from amidine **1** (200 mg, 0.33 mmol) and 2-bromo-1,3-bis(dimethylamino)trimethinium perchlorate **15** (111 mg, 0.36 mmol) according to general procedure 4. Reaction time: 6 h. Purified by column chromatography (EtOAc-hexane = 1:3) to give 203 mg (90%) white amorphous solid. R_f_ = 0.48 (EtOAc-hexane = 1:2); [α]_D_ = +69 (c 0.17, CH_2_Cl_2_); ^1^H NMR (400 MHz, CDCl_3_) δ (ppm): 8.62 (2H, s, H-4, H-6), 7.36–6.86 (20H, m, aromatics), 4.94 (2H, s, Ph*CH_2_*), 4.85, 4.57 (2 × 1H, 2d, *J* = 10.8 Hz, Ph*CH_2_*), 4.64, 4.28 (2 × 1H, 2d, *J* = 11.6 Hz, Ph*CH_2_*), 4.54, 4.48 (2 × 1H, 2d, *J* = 12.2 Hz, Ph*CH_2_*), 4.50 (1H, d, *J* = 9.6 Hz, H-1′), 4.06 (1H, pt, *J* = 9.6, 9.2 Hz, H-2′), 3.89 (1H, pt, *J* = 9.2, 9.1 Hz, H-3′), 3.75–3.67 (4H, m, H-4′–H-6′a,b); ^13^C NMR (100 MHz, CDCl_3_) δ (ppm): 164.3 (C-2), 157.7 (C-4, C-6), 138.6, 138.1, 138.0, 137.9, 128.5–127.5 (aromatics), 119.9 (C-5), 87.3, 82.3, 80.8, 79.9, 78.3 (C-1′–C-5′), 75.7, 75.2, 74.6, 73.5 (4 × Ph*C*H_2_), 69.1 (C-6′). ESI-MS positive mode (*m/z*): Calcd for C_38_H_38_BrN_2_O_5_^+^ [M + H]^+^ 681.1959; C_38_H_37_BrN_2_NaO_5_^+^ [M + Na]^+^ 703.1778. Found: [M + H]^+^ 681.1965; [M + Na]^+^ 703.1782.

*2-(2′,3′,4′,6′-Tetra-*O*-benzyl-β-*D*-glucopyranosyl)-pyrimidine-5-carbaldehyde* (**17d**). Prepared from amidine **1** (200 mg, 0.33 mmol) and 2-dimethylaminomethylene-1,3-bis(dimethylimonio)propane diperchlorate **16** (139 mg, 0.36 mmol) according to general procedure 4. Reaction time: 4 h. Purified by column chromatography (EtOAc-hexane = 1:2) to give 180 mg (86%) white solid. Mp: 80–82 °C; R_f_ = 0.62 (EtOAc-hexane = 1:1); [α]_D_ = +80 (c 0.21, CH_2_Cl_2_); ^1^H NMR (400 MHz, CDCl_3_) δ (ppm): 10.06 (1H, s, CHO), 9.01 (2H, s, H-4, H-6), 7.36–6.84 (20H, m, aromatics), 4.94 (2H, s, Ph*CH_2_*), 4.86, 4.58 (2 × 1H, 2d, *J* = 10.7 Hz, Ph*CH_2_*), 4.65, 4.25 (2 × 1H, 2d, *J* = 11.6 Hz, Ph*CH_2_*), 4.63 (1H, d, *J* = 9.5 Hz, H-1′), 4.54, 4.49 (2 × 1H, 2d, *J* = 12.2 Hz, Ph*CH_2_*), 4.12 (1H, pt, *J* = 9.5, 9.3 Hz, H-2′), 3.92 (1H, pt, *J* = 9.2, 9.1 Hz, H-3′), 3.77-3.69 (4H, m, H-4′–H-6′); ^13^C NMR (100 MHz, CDCl_3_) δ (ppm): 188.8 (CHO), 170.3 (C-2), 158.2 (C-4, C-6), 138.6, 138.1, 138.0, 137.9, 128.6–127.5 (aromatics), 127.7 (C-5), 87.3, 82.7, 81.0, 80.0, 78.3 (C-1′–C-5′), 75.8, 75.3, 74.7, 73.6 (4 × Ph*C*H_2_), 69.2 (C-6′). ESI-MS positive mode (*m/z*): Calcd for C_39_H_39_N_2_O_6_^+^ [M + H]^+^ 631.2803; C_39_H_38_N_2_NaO_6_^+^ [M + Na]^+^ 653.2622. Found: [M + H]^+^ 631.2806; [M + Na]^+^ 653.2626.

*2-(2′,3′,4′,6′-Tetra-*O*-benzyl-β-D-glucopyranosyl)-5-phenylpyrimidine* (**17e**). Compound **17c** (380 mg, 0.56 mmol), phenylboronic acid (136 mg, 1.12 mmol, 2 equiv.), Pd(PPh_3_)_2_Cl_2_ (79 mg, 0.11 mmol, 0.2 equiv.), Cs_2_CO_3_ (363 mg, 1.12 mmol, 2 equiv.), and Bu_4_NF (1.12 mL, 1.12 mmol, 2 equiv., 1M solution in dry THF) were heated at 100 °C in dry 1,4-dioxane (10 mL). After 16 h, the solvent was removed under diminished pressure and the residue was purified by column chromatography (EtOAc-hexane = 1:2). Yield: 340 mg (90%), white amorphous solid. R_f_ = 0.29 (EtOAc-hexane = 1:2); [α]_D_ = +55 (c 0.27, CH_2_Cl_2_); ^1^H NMR (400 MHz, CDCl_3_) δ (ppm): 8.86 (2H, s, H-4, H-6), 7.56–6.87 (25H, m, aromatics), 4.97, 4.94 (2 × 1H, 2d, *J* = 11.1 Hz, Ph*CH_2_*), 4.87, 4.59 (2 × 1H, 2d, *J* = 10.8 Hz, Ph*CH_2_*), 4.65, 4.28 (2 × 1H, 2d, *J* = 11.4 Hz, Ph*CH_2_*), 4.63 (1H, d, *J* = 9.6 Hz, H-1′), 4.56, 4.50 (2 × 1H, 2d, *J* = 12.2 Hz, Ph*CH_2_*), 4.20 (1H, pt, *J* = 9.6, 9.3 Hz, H-2′), 3.93 (1H, pt, *J* = 9.3, 9.1 Hz, H-3′), 3.80–3.72 (4H, m, H-4′–H-6′a,b); ^13^C NMR (100 MHz, CDCl_3_) δ (ppm): 165.0 (C-2), 155.1 (C-4, C-6), 138.7, 138.1 (3), 134.2, 133.2, 129.5–127.1 (aromatics, C-5), 87.3, 82.9, 81.2, 79.8, 78.4 (C-1′–C-5′), 75.7, 75.2, 74.7, 73.5 (4 × Ph*C*H_2_), 69.2 (C-6′). ESI-MS positive mode (*m/z*): Calcd for C_44_H_43_N_2_O_5_^+^ [M + H]^+^ 679.3. Found: [M + H]^+^ 679.6.

*Methyl 2-(2′,3′,4′,6′-tetra-*O*-benzyl-β-D-glucopyranosyl)-pyrimidine-5-carboxylate* (**17f**). To a solution of compound **17d** (100 mg, 0.16 mmol) in dry CH_3_CN (2 mL) NIS (107 mg, 0.48 mmol, 3 equiv.), K_2_CO_3_ (67 mg, 0.48 mmol, 3 equiv.) and MeOH (32 μL, 0.79 mmol, 5 equiv.) were added. The reaction mixture was stirred at rt until the TLC (EtOAc-hexane = 2:3) showed complete transformation of the starting material (5 h). The reaction was then quenched with 10% aq. solution of Na_2_S_2_O_3_ (10 mL) and the mixture was extracted with EtOAc (3 × 10 mL). The combined organic phase was washed with brine (10 mL), dried over MgSO_4_, filtered, and the solvent was removed under diminished pressure. Column chromatographic purification of the residue (EtOAc-hexane = 1:2) gave 72 mg (69%) white amorphous solid. R_f_ = 0.33 (EtOAc-hexane = 1:2); [α]_D_ = +53 (c 0.20, CH_2_Cl_2_); ^1^H NMR (400 MHz, CDCl_3_) δ (ppm): 9.14 (2H, s, H-4, H-6), 7.36–6.84 (20H, m, aromatics), 4.94 (2H, s, Ph*CH_2_*), 4.86, 4.58 (2 × 1H, 2d, *J* = 10.8 Hz, Ph*CH_2_*), 4.61, 4.21 (2 × 1H, 2d, *J* = 11.5 Hz, Ph*CH_2_*), 4.61 (1H, d, *J* = 9.6 Hz, H-1′), 4.55, 4.49 (2 × 1H, 2d, *J* = 12.2 Hz, Ph*CH_2_*), 4.11 (1H, pt, *J* = 9.6, 9.3 Hz, H-2′), 3.99 (3H, s, OCH_3_), 3.91 (1H, pt, *J* = 9.3, 9.1 Hz, H-3′), 3.77–3.69 (4H, m, H-4′–H-6′a,b); ^13^C NMR (100 MHz, CDCl_3_) δ (ppm): 169.4, 164.1 (COOMe, C-2), 158.2 (C-4, C-6), 138.6, 138.1, 138.0, 137.8, 128.5–127.5 (aromatics), 123.1 (C-5), 87.2, 82.7, 8, 1.0, 79.9, 78.3 (C-1′–C-5′), 75.8, 75.2, 74.7, 73.5 (4 × Ph*C*H_2_), 69.1 (C-6′), 52.8 (OCH_3_). ESI-MS positive mode (*m/z*): Calcd for C_40_H_41_N_2_O_7_^+^ [M + H]^+^ 661.3. Found: [M + H]^+^ 661.6.

*2-(β-D-Glucopyranosyl)-pyrimidine* (**18a**). Method A**:** Prepared from amidine **2** (100 mg, 0.41 mmol) and 1,3-bis(dimethylamino)trimethinium perchlorate **13** (103 mg, 0.45 mmol) according to general procedure 4. Reaction time: 16 h. Purified by column chromatography (CHCl_3_-MeOH = 5:1) to give 81 mg (81%) colourless syrup. Method B**:** Compound **17a** (200 mg, 0.33 mmol) was dissolved in anhydrous CH_2_Cl_2_ (10 mL). The stirred reaction mixture was cooled to ‒78 °C and ~1M solution of BCl_3_ in CH_2_Cl_2_ (1.7 mL, 1.7 mmol, 5 eqiuv.) was added. The stirring was continued at this temperature and the reaction was monitored by TLC (EtOAc-hexane = 1:2 and CHCl_3_-MeOH = 3:1). After the complete disappearance of the starting material (3 h), MeOH (15 mL) was added to the reaction mixture and was left to warm to rt. The solvents were removed under diminished pressure and the residue was purified by column chromatography (CHCl_3_-MeOH = 5:1) to give 68 mg (85%) colourless syrup. R_f_ = 0.28 (CH_3_Cl-MeOH = 7:3); [α]_D_ = +44 (c 0.24, H_2_O); ^1^H NMR (360 MHz, CD_3_OD) δ (ppm): 8.84 (2H, d, *J* = 5.0 Hz, H-4, H-6), 7.48 (1H, t, *J* = 5.0 Hz, H-5), 4.44 (1H, d, *J* = 9.6 Hz, H-1′), 3.88 (1H, dd, *J* = 12.2, 1.7 Hz, H-6′a), 3.76 (1H, pt, *J* = 9.5, 9.1 Hz, H-2′), 3.72 (1H, dd, *J* = 12.2, 4.7 Hz, H-6′b), 3.58 (1H, pt, *J* = 9.1, 9.0 Hz, H-3′ or H-4′), 3.52 (1H, pt, *J* = 9.3, 9.1 Hz, H-3′ or H-4′), 3.49–3.46 (1H, m, H-5′); ^13^C NMR (90 MHz, CD_3_OD) δ (ppm): 167.9 (C-2), 158.7 (2) (C-4, C-6), 122.2 (C-5), 83.6, 82.3, 79.3, 74.8, 71.2 (C-1′–C-5′), 62.7 (C-6′). ESI-MS positive mode (*m/z*): C_10_H_14_N_2_NaO_5_^+^ [M + Na]^+^ 265.0795. Found: 265.0795.

*5-Chloro-2-(β-D-glucopyranosyl)-pyrimidine* (**18b**). Prepared from amidine **2** (100 mg, 0.41 mmol) and 2-chloro-1,3-bis(dimethylamino)trimethinium hexafluorophosphate **14** (139 mg, 0.45 mmol) according to general procedure 4. Reaction time: 2 h. Purified by column chromatography (CHCl_3_-MeOH = 9:1) to give 100 mg (88%) white solid. Mp: 200–202 °C; R_f_ = 0.25 (CH_3_Cl-MeOH = 5:1); [α]_D_ = ‒11 (c 0.22, H_2_O); ^1^H NMR (360 MHz, CD_3_OD) δ (ppm): 8.87 (2H, s, H-4, H-6), 4.43 (1H, d, *J* = 9.6 Hz, H-1′), 3.87 (1H, dd, *J* = 12.3, 1.7 Hz, H-6′a), 3.76 (1H, pt, *J* = 9.5, 9.1 Hz, H-2′), 3.70 (1H, dd, *J* = 12.3, 4.8 Hz, H-6′b), 3.54 (1H, pt, *J* = 9.2, 9.0 Hz, H-3′ or H-4′), 3.50–3.43 (2H, m, H-3′ or H-4′, H-5′); ^13^C NMR (90 MHz, CD_3_OD) δ (ppm): 165.9 (C-2), 157.1 (2) (C-4, C-6), 132.1 (C-5), 83.6, 82.6, 79.3, 74.7, 71.4 (C-1′–C-5′), 62.8 (C-6′). ESI-MS positive mode (*m/z*): C_10_H_13_ClN_2_NaO_5_^+^ [M + Na]^+^ 299.0405. Found: 299.0407.

*5-Bromo-2-(β-D-glucopyranosyl)-pyrimidine* (**18c**). Prepared from amidine **2** (100 mg, 0.41 mmol) and 2-bromo-1,3-bis(dimethylamino)trimethinium perchlorate **15** (138 mg, 0.45 mmol) according to general procedure 4. Reaction time: 2 h. Purified by column chromatography (CHCl_3_-MeOH = 9:1) to give 112 mg (85%) white solid. Mp: 224–226 °C; R_f_ = 0.25 (CH_3_Cl-MeOH = 5:1); [α]_D_ = +19 (c 0.22, H_2_O); ^1^H NMR (360 MHz, CD_3_OD) δ (ppm): 8.96 (2H, s, H-4, H-6), 4.41 (1H, d, *J* = 9.6 Hz, H-1′), 3.87 (1H, dd, *J* = 12.2, 1.5 Hz, H-6′a), 3.75 (1H, pt, *J* = 9.6, 9.1 Hz, H-2′), 3.70 (1H, dd, *J* = 12.2, 4.6 Hz, H-6′b), 3.54 (1H, pt, *J* = 9.3, 9.1 Hz, H-3′ or H-4′), 3.50–3.43 (2H, m, H-3′ or H-4′, H-5′); ^13^C NMR (90 MHz, CD_3_OD) δ (ppm): 166.2 (C-2), 159.4 (2) (C-4, C-6), 120.9 (C-5), 83.7, 82.6, 79.3, 74.7, 71.4 (C-1′–C-5′), 62.8 (C-6′). ESI-MS positive mode (*m/z*): C_10_H_13_BrN_2_NaO_5_^+^ [M + Na]^+^ 342.9900. Found: 342.9901.

*2-(β-D-Glucopyranosyl)-5-phenylpyrimidine* (**18e**). Compound **17e** (200 mg, 0.29 mmol) was dissolved in anhydrous CH_2_Cl_2_ (10 mL). The stirred reaction mixture was cooled to ‒78 °C and a ~1M solution of BCl_3_ in CH_2_Cl_2_ (1.5 mL, 1.5 mmol, 5 equiv.) was added. The stirring was continued at this temperature and the reaction was monitored by TLC (EtOAc-hexane = 1:2 and CHCl_3_-MeOH = 3:1). After the complete disappearance of the starting material (2 h), MeOH (10 mL) was added to the reaction mixture and was left to warm to rt. The solvents were removed under diminished pressure and the residue was purified by column chromatography (CHCl_3_-MeOH = 9:1) to give 87 mg (93%) colourless syrup. R_f_ = 0.50 (CH_3_Cl-MeOH = 3:1); [α]_D_ = –31 (c 0.22, H_2_O); ^1^H NMR (360 MHz, CD_3_OD) δ (ppm): 9.07 (2H, s, H-4, H-6), 7.72 (2H, d, *J* = 7.1 Hz, Ph), 7.56–7.46 (3H, m, Ph), 4.49 (1H, d, *J* = 9.5 Hz, H-1′), 3.90 (1H, dd, *J* = 12.1, 1.7 Hz, H-6′a), 3.81 (1H, pt, *J* = 9.5, 9.1 Hz, H-2′), 3.74 (1H, dd, *J* = 12.1, 4.8 Hz, H-6′b), 3.59 (1H, pt, *J* = 9.1, 9.0 Hz, H-3′ or H-4′), 3.53 (1H, pt, *J* = 9.2, 9.0 Hz, H-3′ or H-4′), 3.53–3.51 (1H, m, H-5′); ^13^C NMR (90 MHz, CD_3_OD) δ (ppm): 166.5 (C-2), 156.3 (2) (C-4, C-6), 135.1, 134.9 (Ph, C-5), 130.6 (2), 130.3, 128.1 (2) (Ph), 83.6, 82.5, 79.4, 74.9, 71.3 (C-1′–C-5′), 62.9 (C-6′). ESI-MS positive mode (*m/z*): C_16_H_18_N_2_NaO_5_^+^ [M + Na]^+^ 341.1108. Found: 341.1108.

*Methyl 2-(β-D-glucopyranosyl)-pyrimidine-5-carboxylate* (**18f**). Compound **17f** (200 mg, 0.30 mmol) was dissolved in anhydrous CH_2_Cl_2_ (10 mL). The stirred reaction mixture was cooled to ‒78 °C and a ~1M solution of BCl_3_ in CH_2_Cl_2_ (1.51 mL, 1.51 mmol, 5 equiv.) was added. The stirring was continued at this temperature and the reaction was monitored by TLC (EtOAc-hexane = 1:2 and CHCl_3_-MeOH = 3:1). After complete disappearance of the starting material (2 h), MeOH (15 mL) was added to the reaction mixture and was left to warm to rt. The solvents were removed under diminished pressure and the residue was purified by column chromatography (CHCl_3_-MeOH = 9:1) to give 74 mg (81%) colourless syrup. R_f_ = 0.31 (CH_3_Cl-MeOH = 5:1); [α]_D_ = +51 (c 0.22, H_2_O); ^1^H NMR (360 MHz, CD_3_OD) δ (ppm): 9.29 (2H, s, H-4, H-6), 4.52 (1H, d, *J* = 9.6 Hz, H-1′), 3.99 (3H, s, OCH_3_), 3.88 (1H, dd, *J* = 12.2, 1.7 Hz, H-6′a), 3.78 (1H, pt, *J* = 9.5, 9.1 Hz, H-2′), 3.72 (1H, dd, *J* = 12.2, 4.6 Hz, H-6′b), 3.62–3.47 (3H, m, H-3′, H-4′, H-5′); ^13^C NMR (90 MHz, CD_3_OD) δ (ppm): 171.0 (C=O), 165.1 (C-2), 159.3 (2) (C-4, C-6), 124.8 (C-5), 83.8, 82.5, 79.3, 74.7, 71.2 (C-1′–C-5′), 62.8 (C-6′), 53.3 (OCH_3_). ESI-MS positive mode (*m/z*): C_12_H_16_N_2_NaO_7_^+^ [M + Na]^+^ 323.0850. Found: 323.0851.

### 3.2. Enzyme Assays

The inhibition of rmGPb by the test compounds was investigated with a maximal inhibitory concentration of 625 µM by applying a general protocol described earlier [17,27].

In the glycosidase assays, the inhibition experiments were made under the same conditions, except for buffer composition, substrate and enzyme concentration, which were as follows: 

β-Glucosidase from almonds (Sigma-Aldrich Kft., Budapest, Hungary): 2.5 mM PNP-β-Glc substrate in citrate-phosphate buffer pH 5.2 using 0.25 mg/mL of enzyme.

α-Glucosidase from Saccharomyces cerevisiae (Sigma-Aldrich Kft., Budapest, Hungary): 0.5 mM PNP-α-Glc in glycerophosphate buffer pH 6.9 using 0.02 mg/mL of enzyme. 

Bovine liver β-galactosidase (Sigma-Aldrich Kft., Budapest, Hungary): 1 mM PNP-β-Gal in citrate-phosphate buffer pH 7.3 using 0.12 mg/mL of enzyme.

A 10 µL aliquot for each of the different inhibitor stock solutions was mixed with 370 µL of the buffer and 20 µL of the enzyme stock solution in a plastic UV cuvette. After equilibration at 37 °C for 5 min, a 100 µL aliquot of the substrate stock solution was added. The resulting solutions were thoroughly mixed, and the change in absorbance was followed at 400 nm over 240 s in 2 s intervals using the Parallel Kinetics Analysis program of a JASCO V550 (JASCO Tokyo, Japan) spectrophotometer. Progress curves were plotted and fitted to a straight line. ΔA/min values, proportional to initial rate, were considered to be enzyme activities. In a control experiment, the aliquot of the inhibitor solution was replaced by the same amount of buffer. The initial rate data for the enzymatic substrate hydrolysis in the presence and absence of inhibitor were transferred into percentages of overall inhibition and plotted against the inhibitor concentration in logarithmic scale for IC_50_ determination.

## 4. Conclusions

New representatives of 2-*C*-glycopyranosyl pyrimidines, such as 2-*C*-(β-D-glucopyranosyl)-5,6-disubstituted-pyrimidin-4(3*H*)-ones, 4-amino-2-*C*-(β-D-glucopyranosyl)-5,6-disubstituted-pyrimi-dines, and 2-*C*-(β-D-glucopyranosyl)-5-substituted-pyrimidines were synthesized by ring-closures of *O*-perbenzylated and *O*-unprotected *C*-(β-D-glucopyranosyl)formamidine hydrochlorides with methylenemalonic acid derivatives or vinamidinium salts. The inhibitory activities of the resulting 5-mono- and 4,5,6-trisubstituted pyrimidines were investigated against some glycoenzymes. While none of the new compounds proved to be effective against glycogen phosphorylase and α- and β-glucosidase enzymes, some aryl and/or ester substituted derivatives displayed modest inhibitory potency against bovine liver β-galactosidase.

## Figures and Tables

**Table 1:**
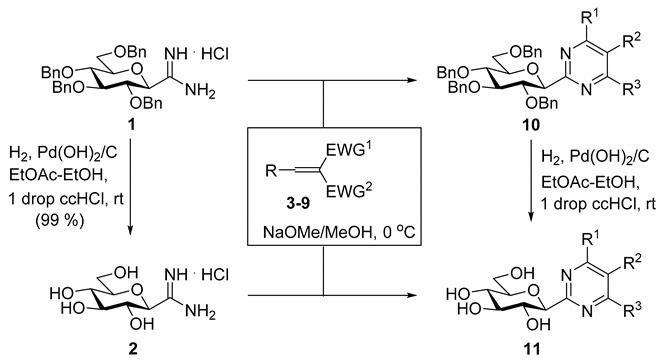
Ring-closure of *C*-(β-D-glucopyranosyl)formamidines with methylenemalonic acid derivatives.

Reagent		Product						
						Yield (%)		
			R^1^	R^2^	R^3^	10	11	
							from 10	from 2
**3**	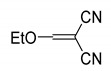	**a**	H	CN	NH_2_	76	n.r. *^a^*	73
**4**	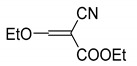	**b**	H	COOEt	NH_2_	37	51	20
		**c**	H	CN	OH *^b^*	30	n.r. *^a^*	45
**5**	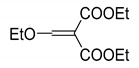	**d**	H	COOEt	OH *^b^*	80	67	51
**6**	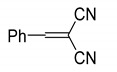	**e**	Ph	CN	NH_2_	78	n.r. *^a^*	85
**7**	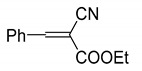	**f**	Ph	CN	OH *^b^*	70	n.r. *^a^*	41
**8**	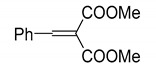	**g**	Ph	COOMe	OH *^b^*	Due to the different outcome of the ring-closure of **1** with dialkyl benzylidenemalonates **8** and **9**, the synthesis of compounds **10g**,**h** and **11g**,**h** are presented separately in Scheme 1.		
**9**	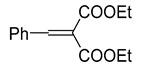	**h**	Ph	COOEt	OH *^b^*			

*^a^* n.r.: No reaction; *^b^* In order to depict compounds **10** and **11** in generalizable chemical formulae, the 6-oxo-1,6-dihydropyrimidine derivatives **10c**,**d**,**f**,**g**,**h**, and **11c**,**d**,**f**,**g**,**h** are shown in their tautomeric 6-hydroxy-pyrimidine forms (R^3^ = OH).

**Table 2:**
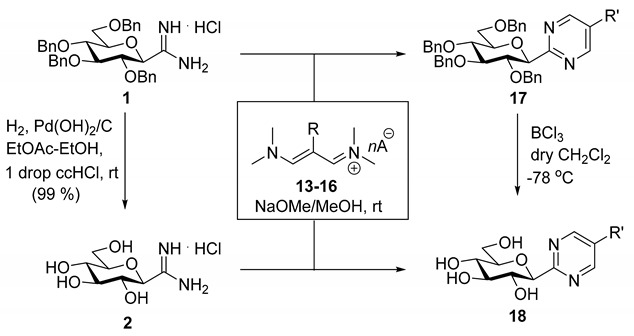
Ring-closure of *C*-(β-D-glucopyranosyl)formamidines with vinamidinium salts.

Reagent				Product				
						Yield (%)		
						17	18	
	R	*n*	A		R’		from 17	from 2
**13**	H	1	ClO_4_	**a**	H	60	85	81
**14**	Cl	1	PF_6_	**b**	Cl	97	ni	88
**15**	Br	1	ClO_4_	**c**	Br	90	ni	85
**16**	CH=NMe_2_^+^	2	ClO_4_	**d**	CHO	86	ni	ni

*ni: not investigated.

**Table 3:** Inhibition of bovine liver β-galactosidase by the new 2-*C*-(β-D-glucopyranosyl)-pyrimidines.

Compound		Inh.	Compound		Inh.
**11a**	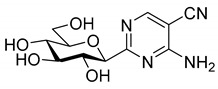	NI at 4.1 mM	**11e**	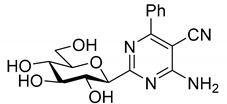	56% at 3.0 mM
**11b**	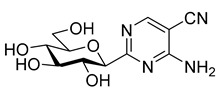	40% at 3.6 mM	**11f**	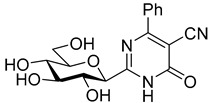	40% at 3.6 mM
**11c**	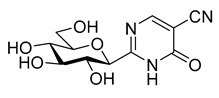	NI at 3.5 mM	**11g**	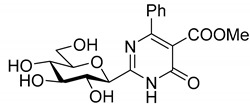	85% at 3 mM
**11d**	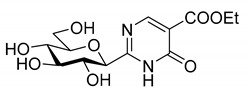	NI at 3.4 mM	**11h**	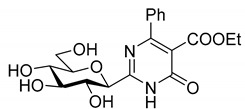	86% at 2.8 mM IC_50_ = 0.294 ± 0.05 mM
**18a**	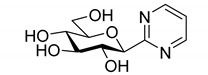	NI at 2.7 mM	**18e**	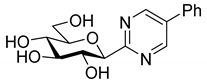	30% at 3.6 mM
**18b**	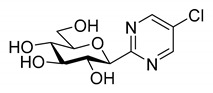	NI at 3.7 mM	**18f**	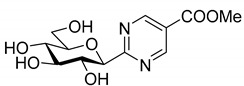	50% at 3.8 mM
**18c**	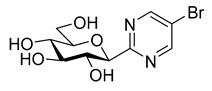	NI at 3.6 mM	**19**	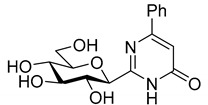	56% at 3.2 mM [17]
